# The Microbiota–Gut–Brain Axis in Light of the Brain Axes and Dysbiosis Where Piezo2 Is the Critical Initiating Player

**DOI:** 10.3390/ijms26157211

**Published:** 2025-07-25

**Authors:** Balázs Sonkodi

**Affiliations:** 1 Department of Health Sciences and Sport Medicine, Hungarian University of Sports Science, 1123 Budapest, Hungary; bsonkodi@gmail.com; 2 Department of Sports Medicine, Semmelweis University, 1122 Budapest, Hungary; 3 Faculty of Health Sciences, Institute of Physiotherapy and Sport Science, University of Pécs, 7624 Pécs, Hungary; 4 Physical Activity Research Group, Szentágothai Research Centre, 7624 Pécs, Hungary

**Keywords:** neurodegenerative disease, Piezo2, channelopathy, enterochromaffin cells, microbiota–gut–brain axis, ultradian rhythm, circadian regulation

## Abstract

The current opinion paper puts into perspective how altered microbiota transplanted from Alzheimer’s patients initiates the impairment of the microbiota–gut–brain axis of a healthy recipient, leading to impaired cognition primarily arising from the hippocampus, dysfunctional adult hippocampal neurogenesis, dysregulated systemic inflammation, long-term spatial memory impairment, or chronic pain with hippocampal involvement. This altered microbiota may induce acquired Piezo2 channelopathy on enterochromaffin cells, which, in turn, impairs the ultrafast long-range proton-based oscillatory synchronization to the hippocampus. Therefore, an intact microbiota–gut–brain axis could be responsible for the synchronization of ultradian and circadian rhythms, with the assistance of rhythmic bacteria within microbiota, to circadian regulation, and hippocampal learning and memory formation. Hippocampal ultradian clock encoding is proposed to be through a Piezo2-initiated proton-signaled manner via VGLUT3 allosteric transmission at a distance. Furthermore, this paper posits that these unaccounted-for ultrafast proton-based long-range oscillatory synchronizing ultradian axes may exist not only within the brain but also between the periphery and the brain in an analogous way, like in the case of this depicted microbiota–gut–brain axis. Accordingly, the irreversible Piezo2 channelopathy-induced loss of the Piezo2-initiated ultradian prefrontal–hippocampal axis leads to Alzheimer’s disease pathophysiology onset. Moreover, the same irreversible microdamage-induced loss of the Piezo2-initiated ultradian muscle spindle–hippocampal and cerebellum–hippocampal axes may lead to amyotrophic lateral sclerosis and Parkinson’s disease initiation, respectively.

## 1. Introduction

Microbiota from Alzheimer’s disease (AD) patients leads to cognitive deficit and impairment of adult hippocampal neurogenesis (AHN) when transplanted into healthy controls, like in the case of brain pathology onset seen in Alzheimer’s disease [1]. Another paper theorized that amyotrophic lateral sclerosis (ALS) is initiated by the progressive irreversible proprioceptive terminal Piezo2 channelopathy-induced loss of forced peripheral oscillatory synchronization to the hippocampal oscillator in an aging-dependent acquired fashion, in addition to environmental risk factors and genetic predisposition [2]. Noteworthy is that recent findings of genetic tracing in regard to ALS are in support of this theory [3,4]. Moreover, this cell autonomous somatosensory terminal Piezo2 channelopathy is proposed to be the primary cause of damage and also suggested to be a principle transcription activator [5]. Accordingly, if acquired, Piezo2 channelopathy is the primary cause of damage, or a common cause of aging onset [6]; then, it should be a common cause of microdamage initiation in neurodegenerative diseases, not only in ALS [7], as supported by genetic variants of unknown significance (VUSs) [3], but also in AD and Parkinson’s disease. The current opinion paper aims to extrapolate this autonomously acquired somatosensory Piezo2 channelopathy-related primary damage theory to the above significant microbiome transfer finding.

## 2. Acquired Piezo2 Channelopathy

Autonomously acquired Piezo2 channelopathy is suggested to take an acute transient, chronic, or irreversible form [5]. This acquired Piezo2 channelopathy is also proposed to cause a switch or miswired proprioception [8], not to mention switches in other pathways, like the metabolic, energy generation, proton-based ultrafast signaling, transcription activation, neuroinflammatory, and pain pathways [8]. Noteworthy is that Piezo2 excitatory stretch and force-gated mechanosensitive ion channels were found to be the principal ones responsible for proprioception [9]. Proprioception is not only the voluntary or involuntary perception of extremities but also underlies a Piezo system, which even involves the somatosensory, neuroinflammatory, neuroimmune, and autonomic regulation of homeostasis in the gut, skin, heart, lung, and eyes, not to mention in the muscles [2,5].

These Piezo2-containing somatosensory nerve terminals contributing to proprioception may endure microdamage, especially when they go through acute excessive hyperexcitational loading under allostatic stress [5]. Acute intensive exercise loading-induced acute stress response (ASR) could be such a moment under prolonged forced lengthening contractions [5]. In support, Piezo2’s role is critical in the defensive arousal response (DAR), as shown in traumatic brain injury (TBI) research [10]. Noteworthy is that mild TBI paralleled delayed-onset muscle soreness (DOMS) is an analogous bi-phasic non-contact injury mechanism on the periphery, evolving from the primary damage of proprioceptive terminals in the form of Piezo2 channelopathy [5,11]. DAR is a critical mechanism in survival and is recalled when an unpredictable threat is perceived by visual and auditory inputs in association with motor abilities [10]. Not only does ASR, which leads to the evolvement of DOMS, share common territories with DAR [5], but exercise also decreases defensive responses to unpredictable threats at higher exercise intensities [12]. This is why the suggested Piezo2 channelopathy of proprioceptive terminals causes microdamage to defensive motor abilities [5], reflected in altered responses to postural perturbations in DOMS [13] and in the significantly increased medium latency response of the stretch reflex [5]. A current submitted manuscript highlighted the ultrafast matching of the Piezo2-initiated eye–brain, auditory/vestibular–brain, and proprioceptive muscle–brain axes within the hippocampal hub [14]. This DOMS-inducing research showed a tendency to mimic a positive Romberg test detected by stabilometry measurements, indicative of proprioceptive impairment [14]. Moreover, the abovementioned TBI research demonstrated via genetic intervention that the restitution of PIEZO2 function reduced escape latency and increased escape speed during DAR [10]. Moreover, this study also showed that neural Piezo2 was the one activating DAR in association with elevated motor abilities [10]. In addition, it was previously theorized that irreversible Piezo2 channelopathy gradually depletes Ca_v_1.3 channel function, especially when VUSs are present in ALS [4], and recently, it was shown in nonhuman primates that Ca_v_1.3 gene therapy indeed results in significant progressive reversal of Parkinson’s disease [15].

Overall, the aforementioned studies support the theorized ultrafast ultradian sensory function of Piezo2 in order to address unpredictable changes, and its acquired channelopathy leads to impaired proprioception and defensive motor abilities [5,14,16].

## 3. The Axes

This proprioceptive terminal Piezo2 microdamage is suggested to impair ultrafast long-range proton-based oscillatory synchronization in the hippocampus [2]. Hence, acquired Piezo2 channelopathy is not only an initiating step towards pathophysiology, presenting the primary damage [5], but reveals the principal importance of the integrity of this underlying ultrafast proton-based neurotransmission backbone of the muscle–brain axis [2]. Correspondingly, if the integrity of the somatosensory terminal Piezo2-induced ultrafast long-range proton-based oscillatory neurotransmission to the hippocampus is this critical underlying backbone of the muscle–brain axis, then these kinds of ultrafast backbone of brain axes should exist elsewhere too.

Hence, the current author proposes the following axes, initiated by Piezo2, within the brain [17], such as the prefrontal cortex–hippocampus axis on which the irreversible Piezo2 channelopathy is suggested by the author to initiate AD pathophysiology in the presence of genetic predisposition and/or environmental risk factors; and the cerebellum–hippocampus axis, on which irreversible Piezo2 channelopathy is suggested by the author to initiate Parkinson’s disease pathophysiology in the presence of genetic predisposition and/or environmental risk factors. These axes operate on an analogous mechanism, like the olfactory bulb–brain communication, theorized to be initiated by Piezo2 [18].

Moreover, the following somatosensory terminal Piezo2-initiated ultrafast long-range proton-based oscillatory synchronizational peripheral–hippocampal axes may also exist, as has been theorized earlier [2,11], such as the muscle–brain axis initiated on type-Ia proprioceptive terminals on which irreversible Piezo2 channelopathy is suggested to initiate ALS pathophysiology in the presence of genetic predisposition and/or environmental risk factors [5]. Additional brain and peripheral–hippocampal axes initiated by Piezo2 are as follows, as theorized by the current author: gut–brain axis initiated by enterochromaffin cells, skin–brain axis initiated by the Merkel-cell neurite complexes or Meissner corpuscles, bone–brain axis initiated by type-Aβ somatosensory fiber terminals, heart–brain axis, lung–brain axis, ear–brain axis, vestibular–brain axis and eye–brain axis from the cornea and retina as well. It is important to note that impairments of these axes are all associated with dysbiosis and autonomic dysregulation [5], not only in AD, but also in ALS [19,20,21] and Parkinson’s disease [22,23]. Acute intensive exercise moments reveal the differential loading of the above somatosensory terminal Piezo2-initiated ultrafast proton signaled brain axes by the regulative control of the autonomic nervous system [2,11]. Crosstalk may exist between somatosensory Piezo2 and autonomic Piezo2 through cross-frequency coupled synchronization, with the involvement of mitochondrial Huygens synchronization [11]. An important finding is the pressure pulse-detection capability of Piezo2 [18], which provides the premise for Piezo2 activation to follow the autonomic regulation and the redistribution of blood flow, for example during acute intensive exercise moments under sympathetic loading. Therefore, the autonomic nervous system and its regulation is the one providing the Janus-faced function of Piezo2 [5]. Consequently, under sympathetic loading different Piezo2 containing somatosensory terminals are activated than under parasympathetic loading, depending on blood flow redistribution. DOMS is suggested to be initiated by such overexcitation moment under forced lengthening contractions and the proprioceptive terminal Piezo2 microdamage evolves under sympathetic loading induced allostatic stress [2,11].

In summary, the Piezo2-initiated underlying ultrafast backbone neurotransmission of the proposed brain axes is provided by the principle ultradian-sensing capability of Piezo2 in order to respond readily to unpredictable changes [5]. This fast-activating and -switching action of Piezo2 may come from its low-frequency Schottky barrier semiconductor diode-like function [11]. It is important to note that enterochromaffin cells (EC) and certain innervating neurons both contain Piezo2; therefore, the low-frequency Schottky barrier semiconductor diode-like function is doubled. It is a loose analogy, but back-to-back connected Schottky diodes behave like one single Schottky contact with lower-barrier height [24]. The current author suggests that this lower-barrier height of Piezo2 activation during the “fight-or-flight” response also instigates a “butterfly in the stomach” feeling, despite a reduction in blood flow to this organ, hence also explaining the abovementioned Janus-faced function [5]. Moreover, it reflects the DAR-associated anticipation-induced activation of the ultrafast ultradian sensory and ultradian rhythm generation function of Piezo2. On the contrary, Piezo2 channelopathy impairs this ultrafast response, as can be observed as a result of inducing DOMS [13] and electrophysiologically represented in the significant delay of the medium latency response of the stretch reflex [5].

## 4. Piezo2 of Enterochromaffin Cells—Microbiome

Parasympathetic loading redistributes the blood flow to a direction that, for example, excites Piezo2 on mechanosensitive EC cells of the colon, which in return induces serotonin secretion from the enteric nervous system. Hence, intact function of Piezo2 activation might be a critical initiating step during parasympathetic loaded redistribution of blood flow [5] and serotonin secretion in the colon; therefore, Piezo2 is also a primary player in the microbiota–gut–brain axis [25]. In support, EC cells are in direct contact with gut microbiota in the colon [25]. Furthermore, mechanosensitive EC cells carry analogous electroexcitatory characteristics through cation channels, like primary sensory cells, and construct a synchronous complex with the enteric nervous system.

Accordingly, the current author theorizes that the low-frequency Schottky barrier semiconductor diode-like feature of Piezo2 [2] on ECs has a role in transducing the circadian oscillatory input of healthy microbiota to the central hippocampal oscillator. In support of this, a circadian clock is suspected in the hippocampus, independent of the suprachiasmatic nucleus, involved in learning and memory [26]. However, the author of this manuscript proposes that the hippocampus also contains an ultradian clock, as part of circadian regulation. It is worthy of note that ultradian rhythm is a biological cycle that is shorter than the 24 h-long circadian rhythm and is repeated episodically more than once within 24 h [27]. Even more importantly, ultradian oscillations are evolutionarily conserved, but their function is far from clear [28]; therefore, the evolutionarily conserved Piezo2 ion channel and its principality may provide an answer for this knowledge gap.

Ultradian oscillations are important in myoblasts, fibroblasts [29,30], embryonic stem cells [31] and neural stem cells where Hes1 expression is the critical player with robust 2–3 h of episodic oscillations [32]. Reactive oxygen species (ROS) drives these ultradian oscillations of Hes1 metabolically [28]. Indeed, a recent paper proposed that, as a result of the microdamaged low-frequency Schottky semiconductor barrier diode-like function of Piezo2, the modulation of ROS-dependent mitochondrial high-frequency oscillations could become malfunctional, leading to increased electron leakage with resultant increased ROS production through the electron transport chain [16]. In addition, the dual role of ROS in hippocampal learning and memory and hippocampal neurotoxicity leading to neurodegeneration is known [33,34], and Hes1 regulates AHN following TBI in order to facilitate the recovery of neural function [35]. Nevertheless, Hes1 also has a function of preventing dysbiosis by regulating gut homeostasis [36] on the other end of the underlying Piezo2-initiated ultrafast ultradian backbone neurotransmission of the microbiota–gut–brain axis. It is noteworthy that Hes1 deletion in gut epithelial cells results in dysbiosis and, e.g., an increase in Escherichia coli in the colon [36]. Further, ROS modulate Hes1 in a calcium-dependent manner [28], and Piezo channels are cation-selective mechanical channels with a slight preference for calcium [37,38]. After all, the author of this opinion piece implicates Piezo2 in control of the initiating modulation of Hes1 expression through ROS. Consequently, Piezo2 channelopathy causes the loss of this control, leading to increased ROS production, dysbiosis and the impairment of AHN along the microbiota–gut–brain axis. Indicative of this role of hippocampal Hes1 is that when its role is promoted with overexpression, Hes1 is protective against amyloid neurotoxicity of neurons [39], which is a critical underlying neuropathology in AD. Accordingly, the current author suggests that proper Piezo2-activation-induced Hes1 expression is essential for homeostatic unfolded protein response (UPR).

Moreover, the link between ultradian rhythm and rapid eye movement (REM) has been long identified [40]. In contrast, non-REM sleep is modulated through serotoninergic infraslow oscillations [41]. Accordingly, the Piezo2-initiated gut–brain axis may provide the long-range proton-based cross-coupled oscillatory synchronization mechanism for the ultrafast integration of EC cells’ Piezo2-initiated ultradian rhythm, with the assistance of microbiota oscillations, to hippocampal circadian regulation and memory formation. Moreover, Piezo1 may contribute to circadian regulation as a diurnal sensor and rhythm generator [16]. By contrast, oscillatory serotoninergic enteric neurons, as part of the enteric nervous system interacting with ECs, provide the slower circadian rhythm domain of the gut–brain axis. The Piezo2-initiated domain of the gut–brain axis synchronizes during REM sleep, fully in line with earlier theories [11], while the serotoninergic domain of the gut–brain axis synchronizes during non-REM sleep. This is one way in which we are tuned to the cyclic environment of the Earth and adjusted to ultradian events, and it should not be forgotten that mitochondria have a bacterial origin. So, the acquired Piezo2 channelopathy may be the underlying reason, according to the current author, why disturbances in REM sleep could indicate neurodegenerative changes and AD progression [42].

In support, some gut bacteria show rhythmic oscillations tuned to the circadian clock, called rhythmic bacteria. These rhythmic bacteria represent around 10–15% of the entire gut microbial bacteria content [43,44]. One recent study theorized that these rhythmic bacteria may contribute to dysbiosis at disease onset. Therefore, this was analyzed in six circadian-related diseases, such as type 2 diabetes, hypertension, atherosclerotic cardiovascular diseases, colorectal cancer, inflammatory bowel disease, metabolic syndrome and one non-circadian disease [45]. Indeed, the results showed association between rhythmic bacteria and circadian-related diseases. However, this weak link [45] should not be overappreciated. Correspondingly, this study should be considered suggestive of a more complex picture in which circadian disruptions are not solely affected by these rhythmic bacteria on the route to disease onset [45]. It is important to note that rhythmic bacteria were highly underrepresented among the most abundant bacteria in this analysis [45]. Additionally, this study also argued that these bacteria were affected by other physiological stressors as well, not only by circadian disruption [45].

**Figure 1:**
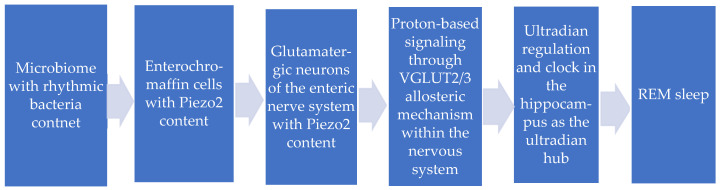
The novel ultradian oscillatory synchronization mechanism from rhythmic bacteria even to rapid eye movement (REM) sleep.

The author of this opinion piece also proposes that this Piezo2-initiated proton-based neurotransmission and ultradian clock encoding is through vesicular glutamate transporter 3 (VGLUT3) allosteric transmission at a distance (see Figure 1). This is in contrast to VGLUT1 and VGLUT2 allosteric transmission, suggested along the muscle–brain axis [2,11]. Piezo2 channelopathy is theorized to switch this ultrafast proton-based signaling to fast glutamate-based signaling; therefore, it leads to muscle mechanical hyperalgesia eventually along this muscle–brain axis [2,11]. Consequently, Piezo2 channelopathy may also be the underlying reason why VGLUT3 is involved in visceral hyperalgesia of irritable bowel syndrome [46] along the gut–brain axis. Moreover, since enteric glutamatergic interneurons regulate intestinal motility through VGLUT2 [47], Piezo2 channelopathy may also cause dysmotility in irritable bowel syndrome through VGLUT2 disruption.

Off note, proton tunneling for Piezo2 arises along the electron transport chain of high-frequency oscillating mitochondria due to oxidative phosphorylation (OXPHOS) [8]. The aforementioned low-frequency Schottky barrier semiconductor diode-like feature of Piezo2 expedites this proton tunneling to extracellular territories, and VGLUT is the critical player in the proposed quantum mechanical allosteric transmission mechanism [5,11].

Correspondingly, it is not only glutamine, short-chain fatty acids (SCFA) or lipopolysaccharide (LPS) production capability of the microbiome that may count [25], but its oscillation in association with the suggested ultradian clock and circadian regulation as well. Therefore, proton affinity might carry high relevance in the suggested Piezo2-initiated pacing of the ultradian clock and circadian regulation of humans. Noteworthy is that not only is circadian regulation hierarchical within humans, but mechanotransduction and energy generation are as well in an interdependent way [5]. Correspondingly, it is suggested that Piezo2 is the principal coupling and synchronization initiator of these hierarchies at ultradian domains within the nervous system through the aforementioned novel ultrafast proton-based cross-frequency coupled oscillatory synchronizational signaling [5]. Moreover, the current author also proposes that the hippocampus is the ultradian clock and ultradian integrative hub for these hierarchies and the aforementioned underlying backbone ultrafast hippocampal axes of the brain axes.

Furthermore, the contribution of the single-stranded RNA content of the microbiome [25] should not be excluded as an ultradian and circadian oscillator either. It is noteworthy that Sugisawa et al. found earlier that Piezo1 of ECs are RNA sensors [48], but Nickolls et al. showed conflicting results [49]. However, the current author suggests that Piezo2-containing oscillatory glutamatergic enteric neurons, as part of the enteric nervous system interacting with Piezo2-containing ECs and with Piezo1 through Piezo2–Piezo1 crosstalk, are not factored in as confounders of unaccounted Piezo2-related cross-frequency-coupled oscillatory synchronizational signaling [11] along the microbiota–gut–brain axis.

In addition, dysbiosis towards high proton affinity may not be preferential in the microbiome, for the aforementioned Piezo2 mechanotransduction in EC cells, because it could impair proton availability for the aforementioned low-frequency Schottky barrier semiconductor diode-like function of Piezo2 [8,11], leading to impaired transduction of microbiome oscillations. Another important consideration is that SCFA, especially SCFA produced by rhythmic bacteria [45], and LPS from the microbiota are essentially involved in transmembrane lipid raft maintenance through the synthesis of lipids, among others, like phospholipase-C, phospholipase-D and PIP2 [2,11]. These lipid rafts are in the close vicinity of Piezo2, and their negative charge is suggested to be crucial in proton availability for Piezo2 [2,11], not to mention their direct modulatory role in Piezo ion channels.

After all, it is worthy to consider that not only the force-from-lipid or force-from-filament principle may count in force-gated Piezo2 activation and modulation, but force-from-proton as well. An important aspect of this consideration is the proposed neuroeconomics of proprioception. This means that neuro-energetic resources of proprioception have a resource limitation under prolonged intensive modulation or, even more importantly, under allostatic stress. Piezo2 channelopathy-induced proprioceptive switch requires more neural circuit-based involvement segmentally in order to compensate for the referred microdamage. Hence, more neuro-energetic resources are needed at the affected segmental level, but this compensation for neuro-energetic resources must be reallocated from a locus in which it is abundantly available due to the aforementioned resource limitation. Further theoretical indication is that one consequence of Piezo2 channelopathy is autonomic dysregulation [2], as was demonstrated in DOMS [5,16]. The consequence of this Piezo2 microdamage is an abrupt neuro-energetic resource reallocation challenge within the autonomic nervous system (ANS) as well, due to Piezo2-channelopathy-induced impaired Piezo2–Piezo2 crosstalk [5,16].

An important underlying energy-generating aspect of this neuroeconomics could be glutamine fermentation. Acquired Piezo2 channelopathy may not only impair the low-frequency Schottky barrier semiconductor diode-like feature of Piezo2 function [11], but could impair the intracellular electrochemical proton gradient due to the dissociation of auxiliary proteins from Piezo2 during prolonged forced lengthening or distention under ASR [2,5]. This impaired intracellular proton gradient may switch mitochondrial energy metabolism from evolutionarily superior energy-generating OXPHOS and glutamine respiration pathways to mitochondrial glucose and, even more importantly, to glutamine fermentation pathways [8]. During fast growth, glucose and glutamine respirofermentation may run simultaneously [50]. Cancer and immune cells are such proliferating cells [50], and the current author suggests that ECs, with their high turnover rate, are such cells as well. Hence, glutamine production of the gut microbiome could be an important energy source under constraints; therefore, during excessive demand, this may also lead to dysbiosis and imbalance of the symbiotic co-functioning of ECs’ mitochondria and the microbiota. Consequently, chronic Piezo2 channelopathy could permanently alter the microbiome, for example, not only towards glutamine production shortage, but higher proton affinity, and in return, it may impair proton availability for ECs, and eventually to proton affinity switch-induced acquired Piezo2 channelopathy [5,8]. These events eventually lead to an impaired microbiota–gut–brain axis. In support, certain Escherichia coli strains are capable of proton motive force generation under certain growth stages [51] that may lead to the aforementioned proton affinity switch on the Piezo2 content of EC cells, exhibiting an acquired Piezo2 channelopathy. It has been shown that Piezo1 is essentially involved in forced-induced ATP secretion [52] which may not different in the case of Piezo2, especially when we consider the Piezo2-Piezo1 crosstalk in the given fluid-filled compartment with selective barrier [5], such as the gut. This forced-induced ATP efflux through Piezo activation feeds the ATPase of Escherichia coli on route to proton motive force generation and in return these bacteria strains deplete OXPHOS on EC cells, as OXPHOS is theorized to be depleted transiently in DOMS as well [8], leading to proton affinity switch and Piezo2 channelopathy.

Lastly, the activation of the UPR pathway occurs within homeostasis prior to Piezo2 channelopathy, and it is meant to be a protective mechanism [7]. However, the UPR and protein degradation are in a state of imbalance in ALS as a result of the Piezo2-channelopathy-initiated mechanism [7]. Moreover, this could be the case in AD as well [53]. It seems that Piezo2-channelopathy-induced switch/miswiring promotes an adaptive response to stress within the endoplasmic reticulum, as is implicated in AD [53], and this adaptive mechanism is theorized to be served by ASIC3 ion channels as secondary proprioceptive channels [5]. Indicative of this is that Piezo buffers mechanical stress even in Drosophila through the modulation of intracellular calcium handling, while the functional mutation of PIEZO miscarries this mechanical stress buffering, leading to pathological remodeling [54]. This is further in support of the fact that Piezo2 is the principal ultradian sensor and even stress buffer; however, the irreversible functional channelopathy of it may lead to a pathological remodeling problem in ALS [2], and this is the case in AD as well [55].

One more symptom of acquired Piezo2 channelopathy is not implicated in the microbiota transfer finding from AD patients, namely insulin resistance induced by the proprioceptive neural switch due to the impairment of Piezo2–Piezo1 crosstalk [8]. In support of this is that an insulin-like growth factor 1 (IGF-1)–Piezo2 pathway exists in the dorsal root ganglion [56]. However, the current author highlights that this pathway may extend all the way upstream to Piezo2-containing somatosensory terminals, initiated by the Piezo2–MyoD pathway [2,5]. Hence, the acquired Piezo2 channelopathy not only impairs its principal intracellular stress buffer function, but impairs ultrafast insulin regulation through the disruption of the suggested Piezo2/MyoD/Piezo2/IGF-1 pathway and the impairment of Piezo2–Piezo1 crosstalk. After all, it is not accidental that DOMS mimics diabetes in its orthostatic imbalance [5], type 2 diabetes is a circadian-related disease [45], and AD is often called type 3 diabetes [57], where circadian rhythm disruption and stress intersect to form a vicious cycle [58].

In summary, the author of this opinion paper proposes that Piezo2 of ECs and innervating sensory neurons, with a double Schottky barrier semiconductor diode-like function, plays a pivotal role in the transduction of the microbiome’s oscillation and in the induction of ultradian rhythm generation along the microbiota–gut–brain axis. Correspondingly, the channelopathy of these Piezo2 ion channels leads to proton affinity switch, dysbiosis, impairment of the ultradian ultrafast backbone of the gut–brain axis, increased ROS production, dyshomeostasis of energy metabolism, REM sleep disturbance and the impairment of ultrafast insulin regulation.

## 5. Concluding Remarks

This opinion manuscript is meant to explain how the transplanted altered microbiota from AD patients [1] may cause acquired functional Piezo2 channelopathy on ECs. This microdamage initiates the impairment of the microbiota–gut–brain axis with the following potential symptoms: impaired cognition (primarily arising from the hippocampus), dysfunctional hippocampal AHN, dysregulated systemic inflammation, long-term spatial memory impairment on the chronic path or chronic pain with hippocampal-central contribution. Hence, the depicted underlying novel ultrafast proton-based cross-frequency coupled oscillatory synchronizational-signaling mechanism theory initiated by Piezo2 provides the ultradian domain of circadian regulation to spatial encoding of hippocampal learning and memory. It seems imperative to open a new direction of research to analyze the proton motive force-generation capability of bacteria constituents of microbiota, since these strains could entrap and sustain Piezo2 channelopathy on EC cells, likely leading to disease onset, impairment of the microbiota–gut–brain axis and ultradian–circadian regulation.
